# Severity and outcome of COVID-19 disease in patients with allergic rhinitis during the pandemic in Qatar – A preliminary report

**DOI:** 10.5339/qmj.2022.fqac.8

**Published:** 2022-04-01

**Authors:** Sami Aqel, Tayseer Ibrahim, Salma Taha, Hassan Mobayed, Maryam Al-Nesf

**Affiliations:** ^1^Adult Allergy and Immunology Division, Department of Medicine, Hamad Medical Corporation, 3050, Doha, Qatar E-mail: SAqel@hamad.qa

**Keywords:** allergic rhinitis, COVID-19, disease severity

## Abstract

Background: Allergic rhinitis and asthma exacerbation are strongly linked to respiratory viral and bacterial infections. COVID-19 pandemic has raised concerns about the risk of infection and the severity of COVID-19 infection in patients with asthma and allergic rhinitis. However, increasing evidence suggests that atopic disease protects against severe COVID-19 illness owing to the underlying type 2 inflammatory process. Many studies have reported the impact of asthma on COVID-19 disease; however, data on allergic rhinitis are scarce. In this study, we aimed to investigate the severity and outcome of COVID-19 disease in adult patients with allergic rhinitis in Qatar during the first pandemic wave.

Methods: We conducted a retrospective chart review of adult patients with a confirmed diagnosis of asthma and/or allergic rhinitis who had a positive COVID-19 RT-PCR between February 01, 2020, and December 01, 2020. Parameters evaluated included the WHO classification of COVID-19 disease severity as mild, moderate, severe, and critical; COVID-19 disease outcome; and mortality. Patients with allergic rhinitis were defined as those with typical allergic rhinitis symptoms and positive skin prick test or specific IgE to perennial or seasonal inhaled allergens. Only data about patients with allergic rhinitis has been presented in this report.

Results: We screened 97 EMR Cerner records of patients who had the diagnosis code for allergic rhinitis. Nine patients met the inclusion criteria of allergic rhinitis diagnosis; the remaining either had no allergy testing or had negative allergy tests. Seven (77.7%) patients had mild COVID-19, whereas only one (11.1%) patient each had moderate and severe disease. The length of hospital stays for 6 patients ranged from 5–13 days, and the remaining 3 patients were quarantined at home. No reports of critical cases or death were identified. All the patients recovered from COVID-19 with a favorable outcome.

Conclusion: This preliminary data showed that most patients with allergic rhinitis had mild COVID-19 disease. Furthermore, all of them recovered well, similar to the available data from previous studies. A limitation of this study is the small population size.

## Figures and Tables

**Table 1:** Characteristics of 9 patients with allergic rhinitis and COVID-19 disease

**Patient**	**Age (years)**	**Gender**	**Comorbidities**	**Total IgE (kU/L)**	**Inhaled allergens**	**Length of hospital (days)**	**Type of quarantine stay**	**COVID-19 severity**

**1**	37	Male	Diabetes hypertension Chronic rhinosinusitis	751	Aspergillus, DF	11	Quarantine facility	Mild

**2**	46	Male	Chronic rhinosinusitis	111	DF, DP	13	Quarantine facility	Mild

**3**	39	Male	Chronic rhinosinusitis	192	DF, DP		Home quarantine	Mild

**4**	53	Male	Chronic rhinosinusitis Bronchial asthma	600	-	6	In-patient medical ward	Moderate

**5**	36	Male	Chronic rhinosinusitis	793	grass pollens mix, weed pollens, DF, DP	13	Quarantine facility	Mild

**6**	38	Male	Hypertension Chronic rhinosinusitis OSA	121	DF, DP and cockroaches	14	Both in-patient medical ward and Quarantine facility	Severe

**7**	38	Male	Chronic rhinosinusitis	340	(Grass pollens mix, Bermuda grass, weed pollens, aspergillus, DF, DP, cockroaches)	13	Quarantine facility	Mild

**8**	32	Female	Chronic rhinosinusitis	-	DF, DP		Home Quarantine	Mild

**9**	28	Male	Chronic rhinosinusitis	213	NA		Home Quarantine	Mild


DF: Dermatophagoides farinae (American dust mite), DP: Dermatophagoides pteronyssinus (European dust mite), OSA: Obstructive sleep apnea.

